# The fate of concomitant mild mitral regurgitation in aortic insufficiency: A neglected subject

**DOI:** 10.3389/fcvm.2022.1035490

**Published:** 2023-01-20

**Authors:** Hao Xu, Ruiming Guo, Donghai Liu, Suyun Hou, Chenhui Qiao, Xin Zhang

**Affiliations:** Department of Cardiothoracic Surgery, First Affiliated Hospital of Zhengzhou University, Zhengzhou, China

**Keywords:** multiple valve disease, mitral regurgitation, aortic insufficiency, atrial fibrillation, cardiac reverse remodeling

## Abstract

**Objectives:**

Mitral regurgitation (MR) is commonly experienced by patients with aortic insufficiency (AI), and in its mild form, it is considered benign. However, the progression of concomitant mild regurgitation after the aortic valve surgery (AVS) for AI is poorly characterized. The current study aimed to define the long-term outcomes of MR after surgery and identify the risk factors involved in deterioration.

**Methods:**

Patients presenting with moderate/severe AI and concomitant mild MR (*n* = 347) between January 2013 and December 2021 were enrolled. MR grade was assessed by transthoracic echocardiography during the follow-up, and deterioration is defined as an increase in grade to moderate or severe MR from the previous follow-up echocardiography. Analysis of risk factors for early mortality, MR deterioration, and long-term mortality was performed.

**Results:**

A total of 278 patients (84.8%) among 328 survivors had at least one follow-up echocardiography, and complete follow-up occurred for 316 patients (96.3%). Mild MR improved to trivial or none in 194 patients (69.8%), progressed to persistent mild MR for 74 patients (26.6%), and deteriorated for 10 patients (3.6%). Preoperative atrial fibrillation [odds ratio (OR), 23.09; 95% confidence interval (CI), 4.35–122.54] and rheumatic AI (OR, 11.61; 95% CI, 1.26–106.85) were shown to be independent risk factors for MR deterioration by generalized linear mixed analysis.

**Conclusion:**

Progression of concomitant mild MR is rare in patients with AI after AVS. However, rheumatic AI and preoperative atrial fibrillation increase the probability of MR deterioration. Careful follow-up for this cohort of patients is recommended.

## Introduction

Concomitant mitral regurgitation (MR) is found in more than 40% of patients with moderate or severe aortic insufficiency (AI) who receive aortic valve surgery (AVS) (1, 2). Guidelines have recommended positive surgical treatment for concomitant primary and secondary severe MR during AI surgery (3), but controversy remains over treatment strategies for concomitant moderate MR (1, 4–6). For a long time, concomitant mild MR in AI was considered benign with no clinical significance, which never seemed to be a question needing concern. However, few clinical data are available to indicate the fate of concomitant mild MR after AVS for AI, leaving the management of concomitant mild MR uninformed.

The current study investigated the fate of concomitant mild MR after AVS for AI to determine the risk factors for MR deterioration. We hypothesized that a few patients with definable characteristics would have MR progression after surgery during long-term follow-up.

## Materials and methods

### Patient selection

This study was approved by the institutional review board of The First Affiliated Hospital of Zhengzhou University. Informed consent was waived due to the retrospective and observational nature of this study. All patients who underwent the aortic valve procedure between January 2013 and December 2021 were reviewed in an electric medical record system. The inclusion criteria were as follows: (1) patients who underwent the aortic valve procedure (including isolated aortic valve replacement, David procedure, Wheat's procedure, and Bentall procedure) having moderate or severe AI; and (2) patients who had preoperative mild MR; and (3) patients of age ≥ 18 years. The exclusion criteria were as follows: (1) concomitant infective endocarditis; (2) concomitant mitral valve replacement during operation; (3) concomitant aortic dissection; (4) concomitant congenital cardiac defect; (5) concomitant more than moderate aortic or mitral stenosis; and (6) previous cardiac operation history. A total of 3,527 patients underwent the aortic valve procedure in our cardiac center between January 2013 and December 2021. According to including/exclusion criteria, 347 patients were enrolled in this study.

### Data collection and echocardiography measurement

The baseline characteristics included age, gender, history of hypertension, history of diabetes, preoperative chronic renal failure, preoperative New York Heart Association (NYHA) classification, and preoperative atrial fibrillation (AF). Echocardiographic data included left ventricular ejection fraction (LVEF), interventricular septal thickness (IVS), right ventricular end-diastolic diameters (RVEDD), left ventricular end-diastolic diameters (LVEDD), left ventricular end-systolic volume (LVESV), left ventricular end-diastolic volume (LVEDV), left atrial diameter (LAD), the aortic diameter which indicates the maximum diameter of the aortic root and ascending aorta, and the degree of mitral and aortic regurgitation. Assessment of valve regurgitation was based on regurgitant area and regurgitant fraction. Concomitant MR is classified as none or trivial (regurgitant area ≤ 1 cm^2^), mild (1 cm^2^ < regurgitant area ≤ 4 cm^2^), moderate (4 cm^2^ < regurgitant area ≤ 8 cm^2^), and severe (8 cm^2^ < regurgitant area). All the echocardiography measurement is conducted based on standard criteria (7). The deterioration of MR is defined as an increase of the MR grade to moderate or severe from the previous follow-up echocardiography.

### Definition and follow-up evaluation

A death that occurred within 1 month after surgery was considered early mortality. Follow-up was conducted through telephone inquiry, internet inquiry, and regular outpatient clinic visits. Until May 2022, 316 patients (96.3%) had at least one follow-up evaluation and 278 patients (84.8%) had at least one echocardiography evaluation during the follow-up.

### Concomitant mitral valvuloplasty and CABG

There were 16 patients (4.6%) and 25 (7.2%) patients undergoing concomitant mitral valvuloplasty and CABG, respectively, in this study. Due to the retrospective nature of this study, the exact reason why concomitant mitral valvuloplasty was conducted for preoperative mild mitral regurgitation was unclear. Among the 16 patients with concomitant mitral valvuloplasty, 5 patients had rheumatic AI and 11 patients had non-rheumatic AI. All the mitral valvuloplasties were performed with the use of a mitral annuloplasty ring. Two patients presented deterioration of MR during the follow-up. All the coronary angiograms and echocardiography were reviewed for patients who underwent concomitant CABG. A coronary artery lesion was found accidentally in the routine preoperative examination and no distinct abnormal ventricular motion was observed.

### Statistical analysis

Continuous variables were presented as median and 75% range, categoric variables were presented as frequency and percentage. The Kolmogorov–Smirnov method was used for the tests of normality. A univariate analysis was performed using the Mann–Whitney *U* test for continuous variables. The logistic regression model was built to verify the risk factors for early mortality. Meanwhile, a logistic regression analysis was used to evaluate the risk factors for the deterioration of MR during the follow-up. The follow-up time was considered a covariate in the analysis. We also performed the generalized linear mixed model to verify the risk factors for the deterioration of MR. Considering the variability among individuals in this study, the individual subject was regarded as a random factor, and preoperative AF and etiology of AI with follow-up time as fixed factors. The covariates with *P* < 0.15 in the univariable analysis were enrolled into the multivariable analysis. A Cox proportional hazards regression analysis was performed to verify the risk factors for long-term survival. The Kaplan–Meier method was used to estimate survival. Collinearity and proportional hazards assumption was tested for the application of the logistics regression analysis and the Cox regression analysis. All presented *P*-values were 2-sided and a *P*-value of < 0.05 was considered statistically significant. All statistical analysis was performed with IBM SPSS Statistics, version 26.0 (IBM Corp, Armonk, NY), jamovi, version 2.0, and R version 4.2.0 (R Project for Statistical Computing, Vienna, Austria) for Mac.

## Results

### Baseline characteristics and surgical data

A total of 347 patients with a median age of 53 years, of which 78.7% were men, were enrolled. AI etiology was classified as rheumatic (2.9%) or non-rheumatic (97.1%) based on the history of rheumatic fever, echocardiographic imaging, and pathology results. Moderate AI was present in 9.5% and severe AI in 90.5% of patients on admission. Concomitant mitral valvuloplasty was performed on 4.6%, 40.1% underwent concomitant aortic procedure, and 7.2% concomitant CABG procedure. Baseline characteristics are presented in Table 1 and Supplementary Table E1.

**Table 1:** Baseline characteristics and preoperative echocardiography.

**Variables**	**Median (25 percentile, 75 percentile)/Number (Frequency)**
Age (year)	53 (45, 62)
Gender (male)	273 (78.7%)
Hypertension	149 (42.9%)
Diabetes	9 (2.6%)
Chronic renal failure	1 (0.3%)
**NYHA classification**	
I	25 (7.2%)
II	18 (5.2%)
III	194 (55.9%)
IV	110 (31.7%)
LVEF (%)	57.0 (48.5, 62.0)
IVS (mm)	10.0 (9.0, 12.0)
RVEDD (mm)	16.0 (15.0, 17.0)
LVEDD (mm)	65 (60.0, 72.0)
LVESV (ml)	104.0 (78.0, 146.0)
LVEDV (ml)	240.0 (188.5, 309.5)
LAD (mm)	40.0 (36.0, 44.0)
Aortic diameters (mm)	43.0 (38.0, 50.0)
**Degree of AI**	
Moderate	33 (9.5%)
Severe	314 (90.5%)
**Etiology of AI**	
No- rheumatic	337 (97.1%)
Rheumatic	10 (2.9%)
**Degree of TR**	
Non-TR	235 (67.7%)
Mild	101 (29.1%)
Moderate	10 (1.9%)
Severe	1 (0.3%)
Atrial fibrillation	18 (5.2%)

AI, aortic insufficiency; IVS, interventricular septal thickness; LAD, left atrial diameter; LVEDD, left ventricular end-diastolic diameters; LVEDV, left ventricular end-diastolic volume; LVEF, left ventricular ejection fraction; LVESV, left ventricular end-systolic volume; NYHA, preoperative New York Heart Association; RVEDD, right ventricular end-diastolic diameter. TR, tricuspid regurgitation.

### Risk factors for early mortality

**Figure 1:**
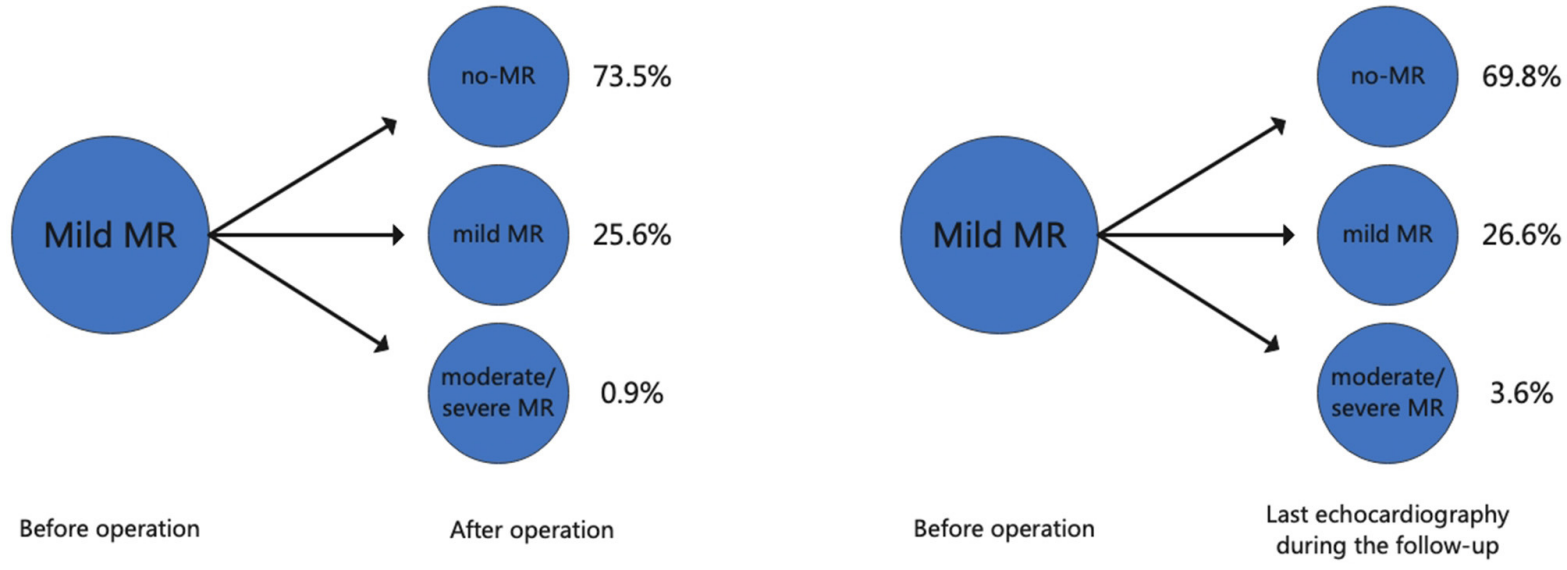
The changes in the degree of mitral regurgitation after aortic valve surgery and during the follow-up. MR, mitral regurgitation.

A total of 19 cases of early mortality (5.5%) occurred among the current cohort and were attributed to 18 cases of cardiac dysfunction (94.7%) and one case of hemorrhagic stroke (5.3%). Evaluation by echocardiography was performed for the 328 patients prior to discharge. MR disappeared post-surgery in 241 (73.5%) patients, persisted in a mild form in 84 (25.6%), and worsened in two patients (Figure 1). The univariate regression analysis showed that older age (OR, 1.07; *P* = 0.006), concomitant mitral valve procedure (OR, 2.48; *P* = 0.102), atrial fibrillation radiofrequency ablation (AFRA) (OR, 7.48; *P* = 0.005), and concomitant aortic arch procedure (OR,38.47; *P* = 0.003) were the risk factors for early mortality. However, the multivariate analysis showed that age (OR, 1.06; *P* = 0.020) and concomitant aortic arch procedure (OR, 39.72; *P* = 0.019) were the only two independent risk factors for early mortality (Table 2).

**Table 2:** Logistic regression analysis for early mortality.

**Variables**	**OR**	**95% CI**	***P***	OR^*^	95% CI^*^	***P***
Age (year)	1.07	(1.02, 1.13)	0.006	1.06	(1.01, 1.12)	0.020
Gender (male)	1.76	(0.65, 4.81)	0.267			
LVEF (%)	0.99	(0.95, 1.04)	0.871			
RVEDD (mm)	0.93	(0.78, 1.12)	0.456			
LVESV (ml)	1.00	(0.99, 1.01)	0.913			
LAD (mm)	0.97	(0.91, 1.04)	0.360			
Degree of AI	0.89	(0.20, 4.02)	0.877			
Etiology of AI	1.97	(0.23, 16.40)	0.531			
Degree of TR	1.43	(0.68, 3.04)	0.347			
Atrial fibrillation	2.29	(0.49, 10.80)	0.293			
Hypertension	0.60	(0.22, 1.61)	0.308			
NYHA classification	0.77	(0.46, 1.31)	0.33			
Mitral valve procedure	2.48	(0.83, 7.38)	0.102	1.87	(0.49, 7.12)	0.356
AFRA	7.48	(1.81, 30.89)	0.005	3.94	(0.65, 23.74)	0.135
Aortic root procedure	0.93	(0.61, 1.42)	0.74			
Aortic arch procedure	38.47	(3.32, 445.57)	0.003	39.72	(1.83, 861.63)	0.019
CABG procedure	1.16	(0.52, 2.62)	0.716			
Tricuspid valve procedure	3.74	(0.76, 18.43)	0.105	0.91	(0.11, 7.60)	0.932
ACCT (minutes)	1.01	(0.99, 1.02)	0.349			
CPB (minutes)	1.01	(0.99, 1.02)	0.055	1.00	(0.99, 1.01)	0.815

ACCT, aortic crossclamp time; AFRA, atrial fibrillation radiofrequency ablation; AI, aortic insufficiency; CABG, coronary artery bypass grafting; CI, confidence interval; CPB, cardiopulmonary bypass; LAD, left atrial diameter; LVEF, left ventricular ejection fraction; LVESV, left ventricular end-systolic volume; NYHA, preoperative New York Heart Association; OR, odds ratio; RVEDD, right ventricular end-diastolic diameter. TR, tricuspid regurgitation. “^*^” indicates the multivariate analysis.

### Echocardiography data and perioperative and follow-up data

**Figure 2:**
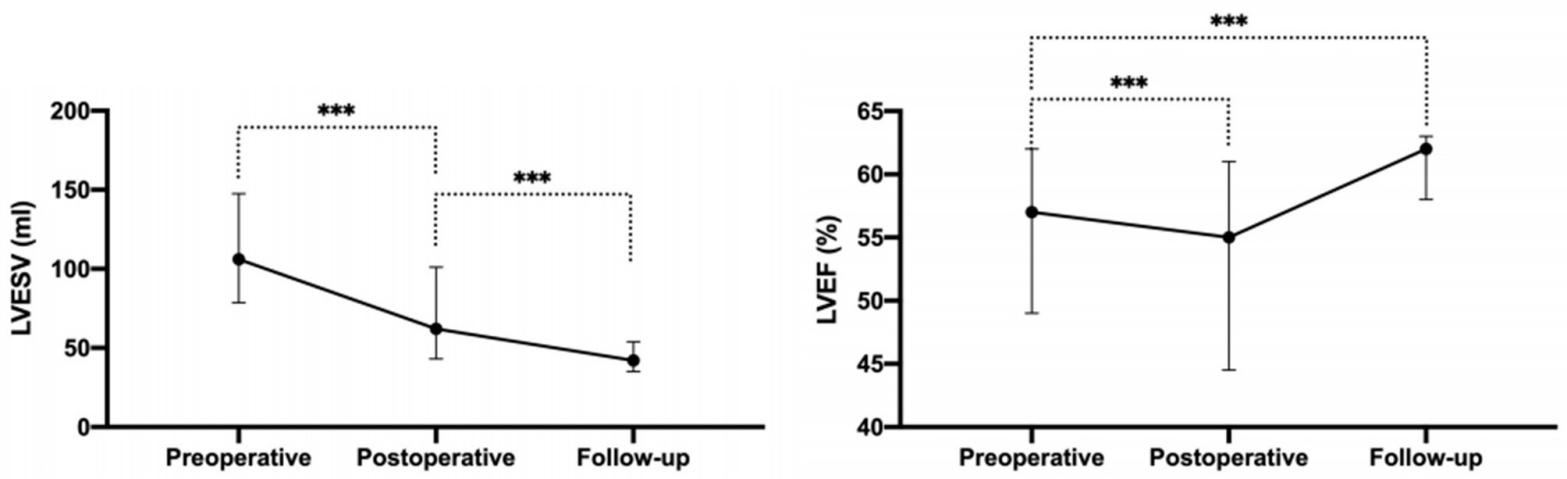
The LVESV and LVEF before operation, after operation, and during the follow-up. LVESV, left ventricular end-systolic volume; LVEF, left ventricular ejection fraction. ^***^P < 0.001.

**Figure 3:**
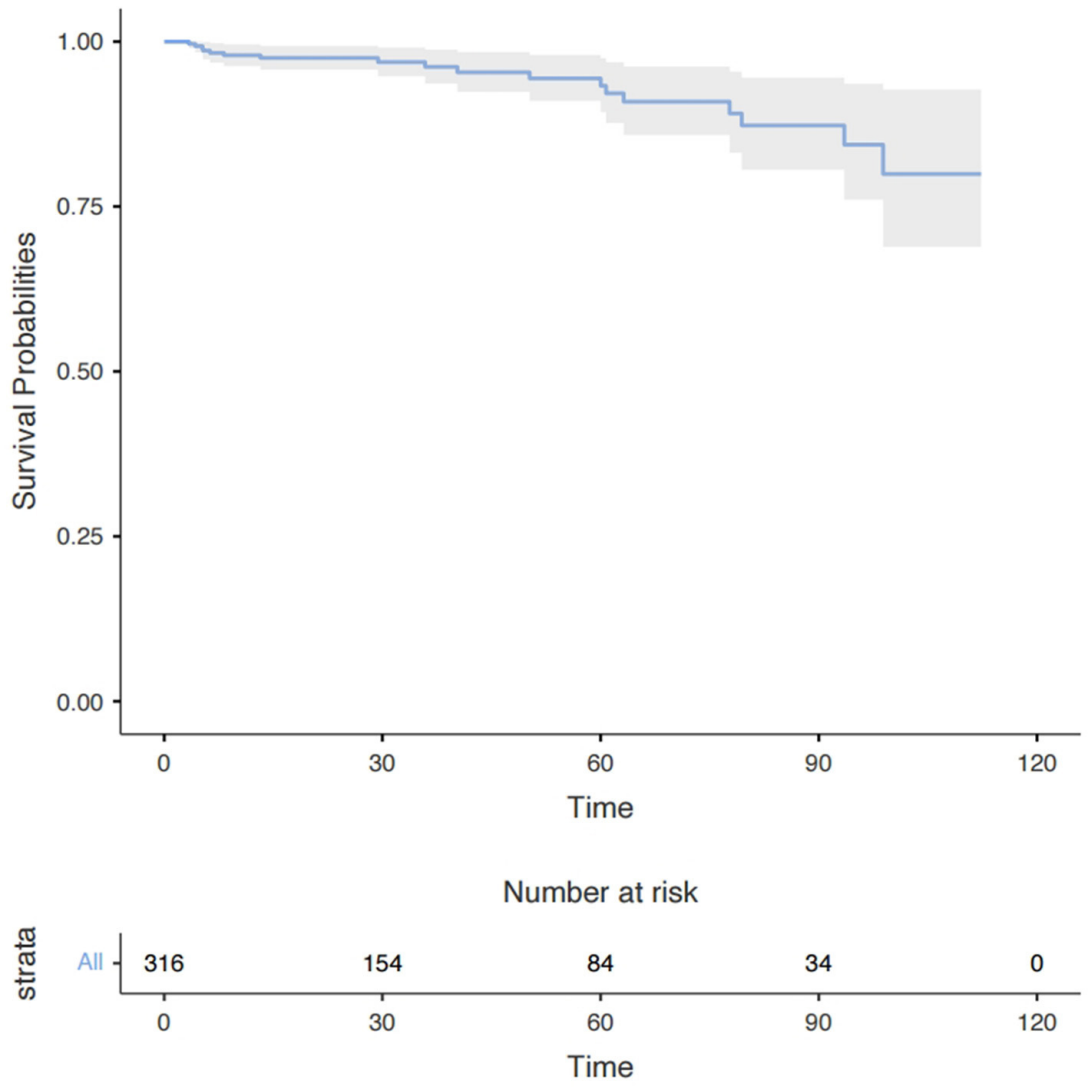
Long-term survival in all patients with moderate AI and concomitant mild MR after the surgery. Data are presented as median with interquartile range. AI, aortic insufficiency; MR, mitral regurgitation.

Complete postoperative and follow-up echocardiography records were available for 273 patients. LVESV was 106.0 ml (79.0, 147.0 ml) before surgery, 62.0 ml (43.0, 101.0 ml) after surgery, and 42.0 ml (35.0, 53.7 ml) during follow-up, demonstrating a gradual decrement (postoperative *vs*. preoperative, *P* < 0.001; follow-up *vs*. postoperative, *P* < 0.001). LVEF values were 57.0% (49.0, 62.0%) before surgery, 55.0% (45.0, 61.0%) after surgery, and 62.0% (58.0, 63.0%) during follow-up (Figure 2). Postoperative LVEF represented a significant decrease from preoperative values (*P* < 0.001). However, follow-up (*P* < 0.001) and postoperative (*P* < 0.001) LVEF values were both greater than the preoperative value. A post-surgical improvement in MR was seen for 241 out of 328 patients. A univariate logistic regression analysis showed that changes in LVESV and LVEF before and after surgery were not related to postoperative MR improvement (LVESV, *P* = 0.613; LVEF, *P* = 0.100). Follow-up echocardiography evaluation of 278 patients (84.8%) showed that mild preoperative MR became trivial or none in 194 (69.8%), mild and persistent in 74 (26.6%), and had deteriorated in 10 patients (3.6%) (Figure 1). A univariate logistic regression analysis showed that moderate AI (OR, 0.19; *P* = 0.024), rheumatic AI (OR, 14.34; *P* = 0.004), preoperative atrial fibrillation (OR, 12.30; *P* = 0.001) (Figure 3), lower NYHA classification (OR, 0.41; *P* = 0.007), less decrement of LVESV during follow-up (OR, 0.98; *P* = 0.013), and longer follow-up time (OR, 1.04; *P* = 0.010) were associated with MR deterioration. However, only rheumatic AI (OR, 61.96; *P* = 0.014), preoperative atrial fibrillation (OR, 93.66; *P* = 0.002), lower NYHA classification (OR, 0.15; *P* = 0.006), and longer follow-up time were the risk factors for MR deterioration during multivariate regression analysis (Table 3). A generalized linear mixed model analysis confirmed preoperative AF (OR, 23.09; *P* < 0.001), rheumatic AI (OR, 11.61; *P* = 0.030), and longer follow-up time (OR, 1.04; *P* = 0.003) to be independent risk factors for MR deterioration during the follow-up (Table 4).

**Table 3:** Logistic regression analysis for deterioration of mitral regurgitation during the follow-up.

**Variables**	**OR**	**95% CI**	**P-value**	OR^*^	95% CI^*^	**P-value**
Age (year)	1.03	(0.97, 1.09)	0.356			
Gender (male)	1.79	(0.43, 7.37)	0.423			
LVEF (%)	1.00	(0.94, 1.08)	0.925			
RVEDD (mm)	1.10	(0.90, 1.36)	0.354			
LVESV (ml)	1.00	(0.99, 1.01)	0.860			
LAD (mm)	1.03	(0.94, 1.13)	0.528			
Degree of AI	0.19	(0.04, 0.80)	0.024	0.50	(0.06, 3.88)	0.504
Etiology of AI	14.34	(2.36, 87.09)	0.004	61.96	(2.31, 1,662.78)	0.014
Degree of TR	1.86	(0.62, 5.54)	0.265			
Atrial fibrillation	12.30	(2.68, 56.42)	0.001	93.66	(5.27, 1,664.12)	0.002
Hypertension	0.16	(0.02, 1.28)	0.084	1.06	(0.15, 7.73)	0.951
NYHA	0.41	(0.21, 0.78)	0.007	0.15	(0.04, 0.57)	0.006
Mitral valve procedure	6.45	(1.21, 34.38)	0.029	1.74	(0.14, 21.14)	0.664
AFRA	4.13	(0.46, 37.18)	0.206			
Aortic root procedure	1.03	(0.60, 1.75)	0.917			
CABG procedure	0.99	-	> 0.05			
Tricuspid valve procedure	3.61	(0.41, 32.02)	0.249			
ACCT (minutes)	0.98	(0.96, 1.01)	0.239			
CPB (minutes)	0.99	(0.97, 1.01)	0.415			
Follow-up time (month)	1.03	(1.01, 1.05)	0.002	1.04	(1.01, 1.08)	0.010
ΔLVESV (ml)	0.98	(0.97, 0.99)	0.013	0.99	(0.97, 1.00)	0.105
ΔLVEF (ml)	1.04	(0.97, 1.12)	0.252			

ACCT, aortic crossclamp time; AFRA, atrial fibrillation radiofrequency ablation; AI, aortic insufficiency; CABG, coronary artery bypass grafting; CI, confidence interval; CPB, cardiopulmonary bypass; LAD, left atrial diameter; LVEF, left ventricular ejection fraction; LVESV, left ventricular end-systolic volume; NYHA, New York Heart Association; OR, odds ratio; RVEDD, right ventricular end-diastolic diameter. TR, tricuspid regurgitation. ^*^indicates the multivariate analysis. “ΔLVESV”, the difference between preoperative LVESV and LVESV from last echocardiography; “ΔLVEF”, the difference between preoperative LVEF and LVEF from last echocardiography.

**Table 4:** Predictors for deterioration of mitral regurgitation in long-term follow-up, based on generalized linear mixed model.

**Variables**	**OR**	**95% CI**	***P***	OR^*^	95% CI^*^	***P***
Atrial fibrillation	22.63	(3.38, 99.21)	< 0.001	23.09	(4.35, 122.54)	< 0.001
Follow-up time (month)	1.04	(1.01, 1.06)	0.002			
Etiology of AI	11.08	(1.79, 68.44)	< 0.01	11.61	(1.26, 106.85)	0.030
Follow-up time (month)	1.03	(1.01, 1.05)	< 0.01	1.04	(1.01, 1.06)	0.003

CI, confidence interval; OR, odds ratio. ^*^indicates the multivariate analysis.

### Long-term outcomes

At least one follow-up evaluation was performed in 316 patients (96.3%) up to the cut-off date of May 2022 with the median follow-up time being 28.5 months and the mean 39.3 months. A Kaplan–Meier analysis showed that 1-, 3-, and 6-year survival rates were 97.7, 93.7, and 80.6%, respectively (Figure 3). Eighteen deaths occurred during follow-up, 7 from an unknown cause, 2 from ischemic stroke, 3 from hemorrhagic stroke, 4 from heart failure, and 2 from aortic dissection. The univariate Cox regression analysis identified older age (HR, 1.07; *P* = 0.006) and larger LAD (HR, 1.08; *P* = 0.026) to be risk factors for long-term mortality but the multivariate analysis showed age (HR, 1.06; *P* = 0.020) was the only independent risk factor (Supplementary Table E2).

### Comment

Multiple-valve disease, attributable to several causes, is common in clinical practice (8, 9), and treatment strategies for concomitant valve disease are always a clinical concern. A concomitant surgical procedure for valve disease may increase surgical risk with very limited to no benefits (4, 10). However, lack of attention to the concomitant valve lesion may allow it to deteriorate during follow-up, increasing the likelihood of re-operation (5, 11, 12). These uncertainties make it important to decide on a reasonable surgical strategy for concomitant valve disease at the first operation and understand the post-surgical fate of concomitant valve lesions. However, only minimal data from related studies are available, and there are insufficient information guidelines for treatment. The current study addressed the neglected question of the long-term fate of concomitant mild MR in patients with moderate or severe AI.

Moderate or severe AI significantly increased the volume load of the left ventricle, leading to left ventricular remodeling and secondary MR (13–15). In addition, the pathogenic factors causing AI may also lead to mitral valve lesions and result in concomitant primary MR (16). The consensus is that concomitant severe MR should be treated surgically during AVS (3) and that concomitant moderate secondary MR may gradually improve or disappear without surgical treatment due to postoperative reverse cardiac remodeling (4, 5, 14). However, little is known about the fate of concomitant mild MR after AVS, often considered a stable benign lesion with no impact on hemodynamics. The present cohort included 96.4% of the patients with unchanged or non-detectable MR at follow-up (Supplementary Table E3), consistent with the consensus. However, what was more unexpected was that 10 out of 278 patients experienced MR deterioration during follow-up. Four had pre-operative AF, and AI was attributed to rheumatic disease in two. Accordingly, the current analysis determined that preoperative AF and rheumatic AI were the risk factors for MR deterioration during follow-up.

**Figure 4:**
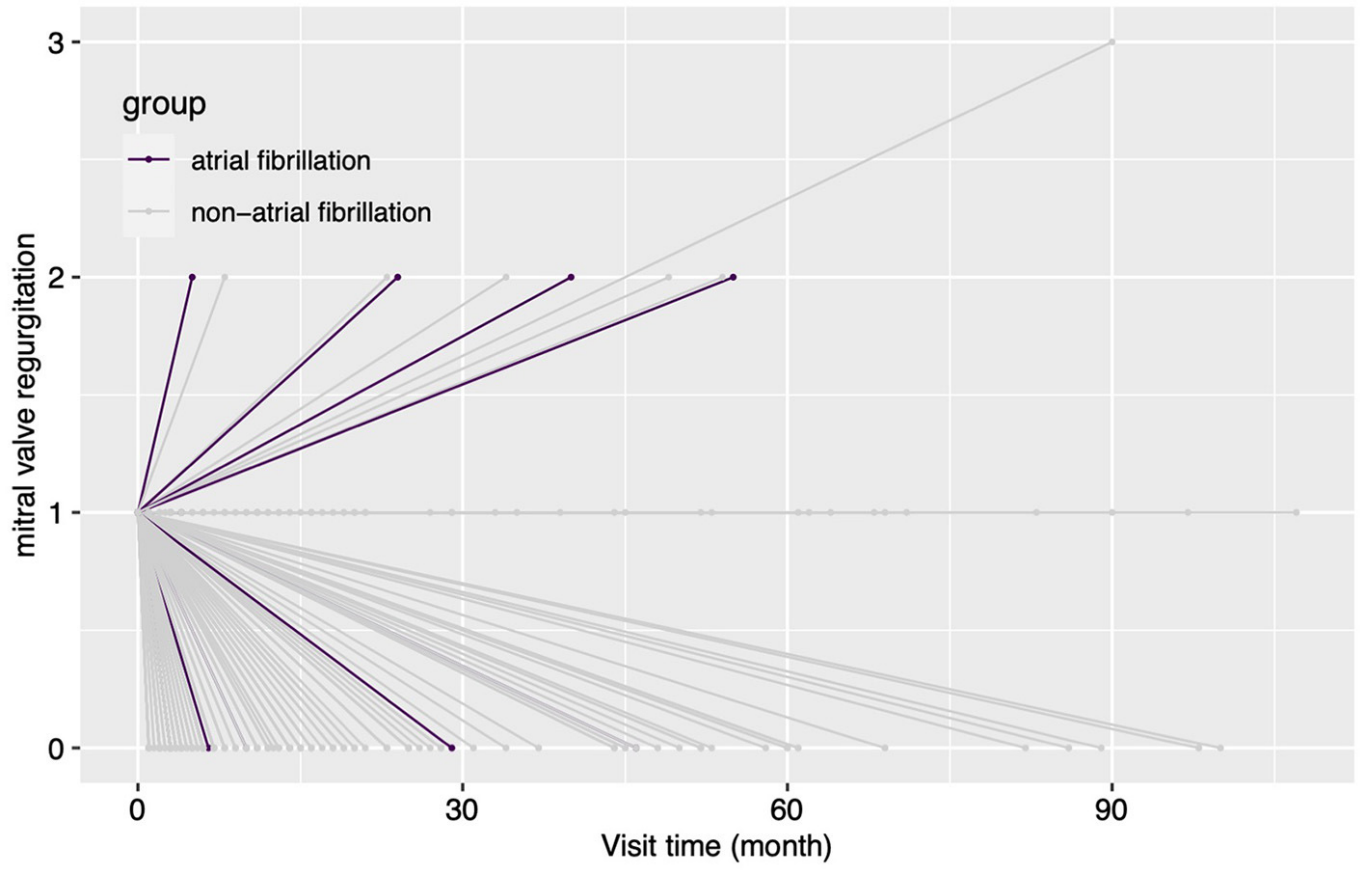
The changes in the degree of MR for patients grouped by concomitant preoperative atrial fibrillation during the follow-up. In this figure, every line indicates the changes in the degree of mitral regurgitation for a single patient, and the two ends of a line indicate the preoperative echocardiography assessment and the last follow-up echocardiography assessment. MR, mitral regurgitation.

**Figure 5:**
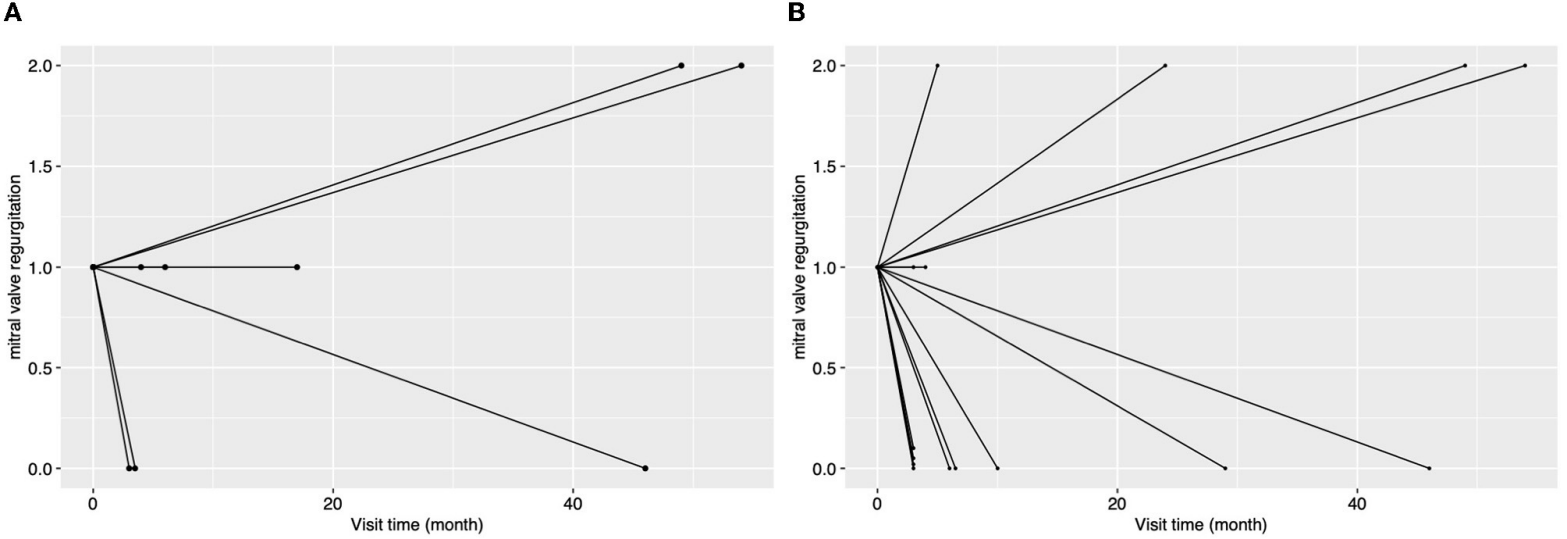
The changes in the degree of MR for patients with rheumatic aortic valve disease [**(A)**, *n* = 8] or with atrial fibrillation [**(B)**, *n* = 15] during the follow-up. In this figure, every line indicates the changes in the degree of mitral regurgitation for a single patient, and the two ends of a line indicate the preoperative echocardiography assessment and the last follow-up echocardiography assessment.

The etiology of concomitant MR has a significant impact on treatment strategy but can be difficult to determine (1). Concomitant secondary MR may improve with post-surgical reverse cardiac remodeling in some conditions (4, 5). However, mild MR may have a complex etiology compounded by several conditions, such as rheumatic disease, mild degenerative lesion, and secondary annulus dilation. The complexity of the influence of MR etiology on MR fate meant that this factor could not be accommodated by the current study. However, an unexpected association between AI etiology and MR fate was found. Concomitant mild MR is more likely to deteriorate when it accompanies rheumatic AI and may be largely due to rheumatic mitral valve lesions, which would not be improved and may worsen after AVS in the long term (11). Preoperative AF was another risk factor for MR deterioration (Figures 4, 5), and this finding may be explained by “atrial functional mitral regurgitation,” (17) when the atrial “pump function” is lost and a high cardiac rate ensues in AF (18). Such factors contribute to atrial remodeling and mitral annulus dilatation, resulting in MR development in the long term. In addition, preoperative AF could be an indicator of atrial remodeling, persistent cardiac overload, and organic mitral valve lesions (18, 19), which would not favor reverse cardiac remodeling and postoperative MR improvement. Longer follow-up time was also associated with a higher possibility of MR deterioration, as might be expected. NYHA classification also correlated with a greater likelihood of MR deterioration, perhaps due to more severe cardiac remodeling and heart failure. Under such conditions, concomitant mild MR may be attributed to functional causes, which would be improved by post-AVS reverse cardiac remodeling. However, no association between reverse remodeling of the left ventricle and MR improvement could be shown. Lim and his colleagues found that postoperative poor left ventricular reverse remodeling was associated with persistent functional MR (4). The same association was not observed during the current study, perhaps due to differences in patient characteristics. Patients of the current cohort all had concomitant primary or secondary mild MR rather than the functional mild to moderate MR of Lim's study.

Age was a risk factor for early and long-term mortality, in agreement with previous studies (20). In addition, concomitant aortic arch procedure increased the early mortality, perhaps due to longer cardiopulmonary bypass (CPB) time, aortic cross-lamp time (ACCT), and the more complex surgical skills required.

### Study limitation

In this study, there are the following limitations. First, this is a retrospective study, which means there are some intrinsic biases, such as treatment bias by different surgeons and recalling bias. Second, the limited number of patients included in this study may reduce the accuracy of the conclusion. In addition, limited numbers of concomitant mitral valve procedures and AFRA in this study made it impossible to explore their roles in the fate of MR in the long term. Finally, due to the retrospective nature of this study and survivor bias, it is impossible to assess the impact of the deterioration of MR on long-term survival, which is an important question for clinicians. Thus, a larger and more prospective study is needed to answer the above questions.

## Conclusion

Patients with moderate/severe AI and concomitant mild MR experience MR deterioration only rarely. Lower preoperative NYHA classification, concomitant AF, and rheumatic AI are risk factors for deterioration in the long term, and patients with these characteristics require careful monitoring of the postoperative state of the mitral valve during follow-up. A larger prospective study should be conducted to confirm the impact of concomitant mitral valve procedure and AFRA on the fate of MR during follow-up and the influence of MR deterioration on long-term survival.

## Data availability statement

The original contributions presented in the study are included in the article/Supplementary material, further inquiries can be directed to the corresponding author.

## Author contributions

Conception and design, data analysis, and interpretation: HX and RG. Administrative support: SH. Provision of study materials or patients and collection of data: HX and DL. All authors wrote the manuscript and approved the final version of the manuscript.
