# Supplementary material for: The fate of concomitant mild mitral regurgitation in aortic insufficiency: A neglected subject
Source: Front Cardiovasc Med. 2023 Jan 20;9:1035490. doi: 10.3389/fcvm.2022.1035490 (PMC9894876; doi:10.3389/fcvm.2022.1035490)
Supplement: Supplementary file 1 [file Table_1.DOCX]

Table E1. Intraoperative Characteristics

| Variables | Median (25 percentile, 75 percentile)/Number (Frequency) |
| --- | --- |
| Mitral valve procedure |  |
| No-procedure | 331 (95.4%) |
| Mitral valvuloplasty | 16 (4.6%) |
| Aortic root procedure |  |
| Non-procedure | 208 (59.9%) |
| Bentall procedure | 72 (20.7%) |
| Wheat’s procedure  Aortoplasty | 19 (5.5%)  35 (10.1%) |
| David’s procedure | 13 (3.7%) |
| Aortic arch procedure | 3 (0.9%) |
| Frozen elephant trunk procedure | 1 (0.3%) |
| AFRA | 11 (3.2%) |
| Tricuspid valve procedure | 12 (3.5%) |
| CABG procedure |  |
| non-graft | 322 (92.8%) |
| 1-graft | 15 (4.3%) |
| 2-graft | 5 (1.4%) |
| 3-graft | 4 (1.2%) |
| 4-graft | 1 (0.3%) |
| ACCT (minutes) | 68.5 (52.0, 91.75) |
| CPB (minutes) | 99.0 (76.3, 131.0) |

ACCT, aortic cross-clamp time; AFRA, atrial fibrillation radiofrequency ablation; CABG, coronary artery bypass grafting; CPB, cardiopulmonary bypass.

Table E2. Cox proportional hazards regression analysis for long-term mortality

| Variables | HR | 95% CI | P | HR* | 95% CI* | P |
| --- | --- | --- | --- | --- | --- | --- |
| Age (year) | 1.07 | (1.02, 1.13) | 0.006 | 1.06 | (1.01, 1.12) | 0.020 |
| Gender (male) | 0.83 | (0.24, 2.88) | 0.773 |  |  |  |
| LVEF (%) | 0.98 | (0.94, 1.03) | 0.428 |  |  |  |
| RVEDD (mm) | 1.10 | (0.98, 1.22) | 0.117 | 1.05 | (0.94, 1.17) | 0.422 |
| LVESV (ml) | 1.00 | (0.99, 1.01) | 0.995 |  |  |  |
| LAD (mm) | 1.08 | (1.01, 1.16) | 0.026 | 1.06 | (0.99, 1.14) | 0.11 |
| Degree of AI | 0.93 | (0.21, 4.05) | 0.919 |  |  |  |
| Etiology of AI | 1.88 | (0.25, 14.25) | 0.541 |  |  |  |
| Degree of TR | 1.47 | (0.66, 3.29) | 0.342 |  |  |  |
| Atrial fibrillation | 3.26 | (0.73, 14.5) | 0.120 | 1.63 | (0.29, 9.07) | 0.58 |
| Hypertension | 1.17 | (0.46, 3.02) | 0.739 |  |  |  |
| NYHA | 1.16 | (0.61, 2.20) | 0.651 |  |  |  |
| Mitral valve procedure | - | - | 0.608 |  |  |  |
| AFRA | 0.20 | (0.03, 1.60) | 0.129 |  |  |  |
| Aortic root procedure | - | - | >0.150 |  |  |  |
| CABG procedure | 0.43 | (0.12, 1.49) | 0.183 |  |  |  |
| Tricuspid valve procedure | 0.30 | (0.04, 2.32) | 0.25 |  |  |  |
| ACCT (minutes) | 1.00 | (0.99, 1.02) | 0.624 |  |  |  |
| CPB (minutes) | 1.00 | (0.99, 1.01) | 0.843 |  |  |  |

ACCT, aortic crossclamp time; AFRA, atrial fibrillation radiofrequency ablation; AI, aortic insufficiency; CABG, coronary artery bypass grafting; CI, confidence interval; CPB, cardiopulmonary bypass; HR, Hazard ratio; LAD, left atrial diameter; LVEF, left ventricular ejection fraction; LVESV, left ventricular end-systolic volume; NYHA, New York Heart Association; RVEDD, right ventricular end-diastolic diameter. TR, tricuspid regurgitation. “*” indicates the multivariate analysis.

Table E3. Echocardiography data during follow-up period

| Variables | All the patients  (n = 278) | Patients with mild or no MR (n = 268) | Patients with worsened MR (n = 10) |
| --- | --- | --- | --- |
| LVEF (%) | 62.0 (58.0, 63.0) | 62.0 (58.0, 63.0) | 60.5 (56.5, 63) |
| RVEDD (mm) | 16.0 (15.0, 18.0) | 16.0 (15.0, 18.0) | 18.5 (16.5, 20.0) |
| LVEDD (mm) | 48.0 (45.0, 52.0) | 47.0 (44.8, 51.3) | 50.5 (49.0, 57.8) |
| LVESV (ml) | 42.0 (35.0, 54.0) | 42.0 (34.8, 53.2) | 51.5 (41.8, 73.0) |
| LVEDV (ml) | 113.0 (94.3, 134.0) | 112.0 (93.0, 134.0) | 121.0 (112.0 170.0) |
| LAD (mm) | 48.0 (45.0, 52.0) | 35.0 (32.0, 39.0) | 42.5 (35.8,47.0) |
| Degree of TR |  |  |  |
| Non-TR | 195 (70.1%) | 192 (71.6%) | 3 (30%) |
| Mild | 71 (25.5) | 68 (25.4%) | 3 (30%) |
| Moderate | 11 (4.0%) | 8 (3.0%) | 3 (30%) |
| Severe | 1 (0.4%) | 0 (0%) | 1 (10%) |

LAD, left atrial diameter; LVEDD, left ventricular end-diastolic diameters; LVEDV, left ventricular end-diastolic volume; LVEF, left ventricular ejection fraction; LVESV, left ventricular end-systolic volume; MR, mitral regurgitation; RVEDD, right ventricular end-diastolic diameter. TR, tricuspid regurgitation.
